# Psychological Resources, Stress, and Well-Being in Adolescence: An Integrative Structural Model

**DOI:** 10.3390/children13010038

**Published:** 2025-12-26

**Authors:** Sándor Rózsa, Andrea Kövesdi

**Affiliations:** 1 Department of Personality and Health Psychology, Institute of Psychology, Károli Gáspár University of the Reformed Church in Hungary, 1091 Budapest, Hungary; 2 Department of Developmental Psychology, Institute of Psychology, Károli Gáspár University of the Reformed Church in Hungary, 1091 Budapest, Hungary; kovesdi.andrea@kre.hu

**Keywords:** adolescence, psychological resources, perceived stress, emotional–behavioral difficulties, health-related quality of life, structural path model

## Abstract

**Highlights:**

**What are the main findings?**

- Psychological resources (self-efficacy, mindfulness, and reflective functioning) were indirectly associated with better health-related quality of life through lower perceived stress and fewer emotional–behavioral difficulties.
- The structural pathways linking resources, stress, difficulties, and HRQoL were highly similar across gender and age groups, and all measures showed good reliability even in 10–12-year-olds.

**What are the implications of the main findings?**

- Interventions aiming to strengthen adolescents’ socio-emotional competences may improve well-being most effectively by targeting stress reduction and emotional/behavioral regulation, not by focusing solely on resource enhancement.
- These psychological measures and frameworks appear suitable for both early and mid-adolescence, supporting timely identification and prevention efforts.

**Abstract:**

**Background/Objectives**: Emotional and behavioral difficulties are common during adolescence and have lasting implications for well-being. Although several psychological resources—such as self-efficacy, mindfulness, and reflective functioning—have been individually linked to better adjustment, less is known about how these strengths jointly relate to perceived stress, difficulties, and health-related quality of life (HRQoL). This study aimed to develop and test an integrative structural model capturing the interplay of these factors during early and mid-adolescence. **Methods**: A cross-sectional online survey was conducted among 395 adolescents (222 girls, 173 boys; aged 10–16 years) who completed self-report questionnaires assessing HRQoL (KIDSCREEN-10), emotional–behavioral difficulties (SDQ), perceived stress (PSS), self-efficacy (GSE), mindfulness (CAMM), and reflective functioning (RFQY-5). After descriptive analyses and correlation testing, the structural path model using observed variables examined how these variables were interrelated. Multi-group analyses assessed whether structural pathways were invariant across gender and age groups. **Results**: Mindfulness, self-efficacy, and reflective functioning were each indirectly associated with better HRQoL, mainly through lower perceived stress and fewer emotional–behavioral difficulties. Perceived stress showed a strong positive association with difficulties, and both constructs uniquely predicted lower HRQoL. The overall pattern of associations was fully consistent across age and broadly comparable across gender. **Conclusions**: The findings highlight the interconnected role of psychological resources, stress, and emotional–behavioral difficulties in adolescents’ well-being. However, the cross-sectional design, convenience sampling, reliance on self-report measures, and single-country sample limit the generalizability and causal interpretation of the results. The robustness of these pathways across age and their broad comparability across gender underscore their developmental relevance and suggest that programs aimed at strengthening socio-emotional competences may be meaningfully applied to support adolescents’ well-being already from early adolescence.

## 1. Introduction

Adolescence is a developmental period characterized by biological, cognitive and social maturation processes that is associated with increased demands on adolescents’ emotion regulation, coping and interpersonal functioning [1]. This period of rapid development represents a sensitive period of the emergence of emotional and behavioral problems, but also an opportunity for the development of psychological resources and socio-emotional competences [2,3]. In this study, mental health is approached as a multidimensional concept that includes emotional, behavioral, and social adjustment, rather than being defined solely by the absence of mental disorders. From this developmental perspective, attention is directed to how adolescents adapt to growing social and academic demands while maintaining their well-being. Although the development of young people’s mental health is influenced by risk factors such as perceived stress or exposure to adverse experiences, protective processes that foster resilience, psychological well-being and adaptive functioning are of equally important significance [4,5].

Socio-emotional competences (SECs) are increasingly seen as a central factor in positive development [6]. They comprise emotion regulation, reflective functioning (i.e., the ability to interpret one’s own and others’ behavior in terms of mental states), self-efficacy, mindfulness, empathy, social problem-solving, and responsible decision-making. Together, these competences enable adolescents to regulate stress, reflect on mental states, build supportive relationships, and feel competent and connected. A number of recent empirical studies have consistently found an association between stronger SECs and lower emotional and behavioral problems and higher prosocial behavior and life satisfaction in adolescents [7,8].

Mindfulness, understood as a non-judgmental awareness in the present moment, has been associated with lower perceived stress and better emotional regulation in children and adolescents [9]. Reflective functioning has been shown to be associated with lower externalising problems, better emotion regulation and more adaptive peer relations [10]. Self-efficacy, i.e., the belief in one’s own ability to cope with challenges, is regarded as an important resilience factor of adolescent development that can predict lower internalizing symptoms and higher life satisfaction [11,12,13].

All of the above variables have been related to the level of youths’ emotional and behavioral adjustment, yet most studies have examined them independently. In real life, adolescents do not rely on a single protective factor but a set of psychosocial resources and competences that interact with each other and shape one’s capacity to cope with stress, to regulate emotions and to respond to difficulties. This highlights the need for an integrative approach that examines the role of multiple protective factors and their interaction with risk processes on well-being.

Perceived stress is one of the strongest predictors of emotional and behavioral problems in children and adolescents [14], and is associated with increased anxiety and depressive symptoms, conduct problems and peer relationship difficulties that jointly threaten well-being and quality of life [15,16]. Youths’ emotional and behavioral difficulties are typically measured with the Strengths and Difficulties Questionnaire (SDQ) [17], while global indices of subjective well-being and functioning are assessed with such instruments as the KIDSCREEN, a widely-used measure of children and adolescents’ health-related quality of life (HRQoL) [18].

Despite the large body of work on these processes, surprisingly few studies have integrated psychological resources, perceived stress, behavioral difficulties and HRQoL in a single structural model. Existing mediation studies typically test only one or two predictors at a time. Key questions remain about how these constructs relate to each other and to functioning and well-being within broader contexts and developmental systems.

A further limitation is that developmental and gender differences have rarely been taken into account in this type of research. Empirical findings suggest that girls experience more internalizing symptoms [19,20] and report more perceived stress than boys. Age-related differences include differences between early and mid-adolescents in cognitive maturity, emotion regulation and sensitivity to peer evaluation [21]. Yet few studies have explicitly tested whether the model structure is invariant across demographic groups. Multi-group SEM is the gold standard for testing such questions, but has been largely overlooked in this field.

Thus, there is a need for research that (a) includes multiple protective and risk processes in a single analytic model, (b) considers the mechanisms linking these processes with HRQoL, a central indicator of global well-being, and (c) tests whether these mechanisms are invariant across gender and developmental stages. Addressing these questions is in line with the scope of this Special Issue.

This study extends previous research by simultaneously modeling multiple psychological resources within a single integrative framework and by testing whether the structural pathways are invariant across gender and age groups. These aspects represent important methodological advances, as such comprehensive, comparative analyses are still rare in adolescent well-being research.

### Research Aims

The study examined how children and adolescents cope with emotional and behavioral challenges during the transition to adolescence. We focused on psychological strengths that help maintain well-being amid growing academic, social, and emotional demands.

A first aim was to specify how general self-efficacy, mindfulness and mentalizing ability, three theoretically distinct but complementary psychological resources, are associated with emotional–behavioral functioning and health-related quality of life (HRQoL). All three are core socio-emotional competences that may foster resilience by strengthening agency, reducing stress reactivity, and supporting the understanding of one’s own and others’ mental states. By incorporating the three predictors into a single structural model, we sought to assess how these psychological resources act as a network rather than in isolation to foster positive psychological functioning during early and mid-adolescence.

Although the study was cross-sectional, a theoretically well-founded model based on previous research on stress and adjustment was tested. We hypothesized that the three psychological resources were indirectly related to HRQoL through the association with perceived stress and emotional–behavioral difficulties, both considered as core processes in adolescent mental health. We explored whether these strengths-based resources could serve as a buffer against the impact of the two core processes on HRQoL and, accordingly, foster a resilient profile of psychological functioning.

We hypothesized a priori that self-efficacy, mindfulness, and reflective functioning would be indirectly associated with adolescents’ health-related quality of life through perceived stress and emotional–behavioral difficulties. In contrast, analyses examining the measurement performance of the scales among 10–12-year-olds and the invariance of structural pathways across gender and age were treated as exploratory questions.

A second aim was to test whether the structural model was the same for the different gender and age groups. It is important to know whether boys and girls, or younger (10–12 years) and older (13–16 years) adolescents, show similar or different pathways, in order to identify which aspects of adjustment are universal and which ones may require more specific support. We applied multi-group path modeling to test whether the relationships among psychological resources, stress, emotional–behavioral problems, and HRQoL were invariant across groups.

Finally, we aimed to investigate the developmental applicability of several commonly used psychological measures. Most self-report instruments have been validated mainly in older adolescents and may be less suitable for assessing emerging socio-emotional competencies in younger age groups. As our sample included 10-year-olds, we examined reliability indices, distributional properties, and age-group differences to evaluate whether these measures performed adequately given early adolescents’ developmental capacities.

## 2. Materials and Methods

### 2.1. Participants and Procedure

Participants were recruited as part of a cross-sectional online survey conducted between February and June 2023. Undergraduate psychology students assisted with recruitment by contacting local schools, youth organizations, and community centers, and by sharing the study link via social media networks. Participants were drawn from different regions of Hungary, including both urban and rural areas, but the sample was not nationally representative. Approximately 520 adolescents (and their parents) were initially invited, and 395 provided usable data, corresponding to an estimated response rate of about 76%.

A total of 395 adolescents participated (222 girls and 173 boys). Their ages ranged from 10 to 16 years (M = 12.77, SD = 1.88), covering the developmental period from late childhood to mid-adolescence. According to information reported by parents and schools, most participants were from middle socio-economic backgrounds. The sample included adolescents from both urban and rural communities, with a larger proportion coming from urban areas.

Participation was voluntary, and no exclusion criteria were applied other than being aged 10–16 years and able to complete the online questionnaire independently. Parental consent was obtained electronically before adolescents were allowed to access the survey, and adolescents provided assent by indicating that they understood the purpose of the study and agreed to take part. The study protocol was reviewed and approved by the Research Ethics Committee of the Institute of Psychology, Károli Gáspár University of the Reformed Church in Hungary (approval number: BTK/476-1/2023).

### 2.2. Measures

#### 2.2.1. KIDSCREEN-10 Index

The KIDSCREEN-10 Index is a brief, internationally validated measure of children’s and adolescents’ health-related quality of life (HRQoL), recommended for use approximately from age 8 to 18 years [18,22]. HRQoL refers to people’s subjective perceptions of their health and well-being in the context of their everyday lives and cultural environment. In line with this definition, the KIDSCREEN-10 provides a global indicator of HRQoL by integrating physical, psychological, and social aspects of functioning into a single, easy-to-interpret score. Throughout this paper, we therefore use the term health-related well-being or HRQoL when referring to KIDSCREEN-10 outcomes, rather than the narrower notion of subjective well-being. The 10 items capture broad components of daily functioning, including general life satisfaction, emotional balance, energy levels, and perceived support from family and peers, rated on 5-point Likert-type scales. Although originally developed as a short form of the longer KIDSCREEN questionnaires, the KIDSCREEN-10 has consistently demonstrated strong psychometric properties across European child and adolescent samples. Internal consistency coefficients in the original validation studies typically ranged between α = 0.78 and α = 0.82 [18], supporting its reliability as a global measure of health-related well-being in both younger and older adolescents. The Hungarian version of the KIDSCREEN questionnaires was also included in the large multinational KIDSCREEN project, which involved extensive coordinated data collection across Europe. The Hungarian subsample exceeded 3000 children and adolescents [22], providing strong support for the psychometric soundness of the instrument in the local context.

#### 2.2.2. Strengths and Difficulties Questionnaire (SDQ)

The Strengths and Difficulties Questionnaire (SDQ) [17] is a widely used behavioral screening tool assessing key aspects of children’s and adolescents’ socio-emotional functioning. The measure consists of five subscales—emotional symptoms, conduct problems, hyperactivity/inattention, peer problems, and prosocial behavior. In the present study, we used the Total Difficulties Score, which is obtained by summing the four difficulties subscales (all except prosocial behavior). The Total Difficulties Score is calculated by summing the four difficulties subscales (emotional symptoms, conduct problems, hyperactivity/inattention, and peer problems), yielding a score between 0 and 40. It functions as a broad indicator of emotional and behavioral problems, capturing both internalizing tendencies (e.g., anxiety, worry) and externalizing features (e.g., impulsivity, conduct difficulties). This use of the Total Difficulties Score is fully consistent with developmental and clinical research, where it is treated as an overall index of child and adolescent mental health difficulties. The score has also been found to be a useful dimensional indicator of psychopathology in clinical and neurodivergent samples [23]. While designed for use in 11–16 year olds, the SDQ is also routinely used in research with younger children and its usage from age 10 is supported by the literature. In the original validation study, Goodman [17] found a Cronbach’s alpha of 0.73 on the Total Difficulties Score, suggesting acceptable internal consistency for use as a global indicator of emotional and behavioral problems. The SDQ has also been widely used and studied in Hungarian samples. The instrument’s reliability and structural validity were found to be acceptable in Hungarian studies and it has been used in community and at-risk youth samples [24,25].

#### 2.2.3. Perceived Stress Scale (PSS)

Perceived stress was measured using the 14-item Perceived Stress Scale (PSS) [26], a widely used instrument assessing the extent to which individuals experience their lives as unpredictable, uncontrollable or overloaded. The items ask respondents to reflect on thoughts and feelings that characterise their subjective experience of stress during the past month, including how often they felt overwhelmed, unable to cope with important things in life, or that they had successfully managed everyday hassles. Responses are given on a 5-point Likert scale ranging from 0 (“never”) to 4 (“very often”), with higher scores indicating higher perceived stress. In the original validation, the PSS achieved Cronbach’s alpha coefficients ranging from 0.74 to 0.91 across three separate samples [26]. In the Hungarian validation, internal reliability was found to be excellent for the 14-item version (α = 0.88), supporting the instrument’s suitability for Hungarian populations [27,28]. Although the PSS-14 is less commonly used in younger early adolescents, we justified its use at age 10 by (a) observing excellent internal reliability in our sample (α = 0.86) and acceptable distributional properties (skewness = 0.15; kurtosis = −0.20; both within ±1), and (b) by noting that the items are worded in a way understandable for late childhood and early adolescence in online self-report formats. Nevertheless, findings related to perceived stress should be interpreted with this age consideration in mind.

#### 2.2.4. General Self-Efficacy Scale (GSE)

General perceived competence was measured with the 10-item General Self-Efficacy Scale (GSE) [29]. The scale reflects individuals’ beliefs about how well they can confront and solve problems, and how likely they are to function well when facing challenging or stressful situations. Items are scored on a 4-point Likert scale ranging from 1 = not at all true to 4 = exactly true, which results in a total score ranging from 10 to 40, with higher scores indicating higher self-efficacy. Even though the GSE was originally designed for use with adults, it has proved to be a reliable and valid measure in adolescent samples as well. A large validation study with more than 1000 high school students in Hungary yielded a Cronbach’s alpha of 0.83 [30], and international reliability coefficients range from 0.76 to 0.90 in different cultural settings [29,31]. The GSE is therefore reliable and appropriate for use with this population.

#### 2.2.5. Child and Adolescent Mindfulness Measure (CAMM)

Mindfulness was assessed using the Child and Adolescent Mindfulness Measure (CAMM) [32], a 10-item self-report instrument developed to capture the degree of non-judgmental, accepting awareness of one’s internal experiences. The CAMM evaluates how openly and flexibly children and adolescents relate to their thoughts, emotions, and bodily sensations—core components of psychological flexibility. Items are rated on a 5-point Likert scale (0 = never true to 4 = always true), and after reverse scoring the positively phrased items, total scores range from 0 to 40. Higher scores indicate greater mindfulness skills. Although originally designed for youth aged 10–17, the CAMM has been widely validated across numerous countries and linguistic contexts. Beyond the English original, the measure has been translated and adapted into Portuguese, Catalan, Dutch, Italian, and French. Across these validations, internal consistency estimates are consistently strong, with Cronbach’s alpha values typically ranging between 0.79 and 0.85, supporting the scale’s reliability in both early and mid-adolescence [33,34,35,36]. The CAMM was translated into Hungarian for the purposes of this study using the standard translation–back translation procedure [37]. The translated version demonstrated good internal consistency (α = 0.84).

#### 2.2.6. Reflective Functioning Questionnaire for Youth (RFQY-5)

Reflective functioning (mentalizing ability) was assessed using the 5-item short form of the Reflective Functioning Questionnaire for Youth (RFQY-5). The RFQY-5 is derived from the longer, 46-item RFQ-Y originally developed to measure how adolescents understand their own and others’ behaviour in terms of underlying mental states—such as feelings, intentions, desires, and beliefs [38]. Unlike the adult RFQ, which contains more abstract and metacognitively demanding items, the youth version uses developmentally appropriate, child-friendly wording specifically tailored to younger respondents. The RFQY-5 items are rated on a 6-point Likert scale ranging from 1 (strongly disagree) to 6 (strongly agree), for a total score ranging from 5 to 30, with higher scores indicating a greater perceived capacity for mentalizing. Although the original RFQ-Y was normed on an adolescent sample aged 11–18 years [39], the brevity and simple language of the 5-item short form make it suitable for use with younger adolescents, as even 10–11-year-olds can understand the items and provide meaningful responses.

Although brief, the RFQY-5 provides acceptable reliability for a multidimensional construct such as mentalizing: Jewell and colleagues [38] obtained a Cronbach’s alpha of 0.75 in their sample of adolescents and found that it can be used as a brief measure of reflective functioning. Although the 5-item form has not yet been specifically validated in Hungary, the full RFQ-Y has recently been adapted and validated in a large Hungarian adolescent sample [40], providing evidence for the broader cultural applicability of reflective functioning measures.

### 2.3. Statistical Analysis

First, we characterized the study variables using descriptive statistics (means, standard deviations, skewness, and kurtosis) and internal consistency estimates (Cronbach’s α). Because the main objective was to test a theoretically informed model, we examined the normality of the observed variables through skewness and kurtosis indices. Following common psychometric recommendations, absolute values below 1.0 were considered acceptable indicators of univariate normality for correlation-based and path analyses [41,42].

To test group differences, we used two-way ANOVAs, with gender (girls, boys) and age group (younger adolescents: 10–12 years; older adolescents: 13–16 years) as between-subjects factors. Due to the slight deviations from normality observed in some variables, we used Spearman’s rank-order correlations to test the associations between all the study variables.

We then estimated a structural path model using observed variables to test the hypothesized relationships among psychological resources (GSE, CAMM, RFQY-5), perceived stress (PSS), emotional–behavioral difficulties (SDQ), and health-related quality of life (KIDSCREEN-10). Following established methodological guidelines [42,43,44,45], model fit was evaluated with multiple indices: χ^2^/df, Comparative Fit Index (CFI), the Tucker–Lewis Index (TLI; [46]), and Root Mean Square Error of Approximation (RMSEA; [47]). All structural models were estimated using maximum likelihood (ML) estimation, which is appropriate given that the observed variables showed near-normal distributions (absolute skewness and kurtosis values < 1).

Conventionally, χ^2^/df < 3, CFI and TLI ≥ 0.95, and RMSEA ≤ 0.06 indicate good model fit, whereas RMSEA values between 0.06 and 0.08 are considered acceptable [43,48].

To assess the developmental stability of the hypothesised pathways, we conducted multi-group structural invariance analyses across gender and age in our sample. This step was important for evaluating whether the same psychological mechanisms underlie stress and well-being across groups [49]. We tested three progressively constrained models:

(a) a configural model, specifying the same overall structure but allowing all parameters to vary freely;
(b) a constrained path model, in which all regression paths were set equal across groups to test structural invariance; and
(c) a residual-constrained model, where the residual variances of the dependent variables were also constrained to equality.

Model comparisons were based on changes in CFI and RMSEA, with ΔCFI ≤ 0.01 and ΔRMSEA ≤ 0.015 indicating invariance [49,50]. Because the analyses were conducted using observed scale scores rather than latent factors, traditional tests of measurement invariance (e.g., loadings, intercepts) were not applicable. Instead, we focused on the invariance of regression paths and residuals across groups.

All the descriptive analyses were conducted with SPSS 22 [51]. We estimated all SEM models with AMOS 23 [52] and JASP (version 0.18).

## 3. Results

### 3.1. Descriptive Statistics and Reliability of the Measures

The descriptive statistics and reliability estimates for all study measures are summarised in Table 1. Overall, the internal consistency coefficients were acceptable across instruments, both in light of international validation studies and, where available, Hungarian adaptation work. All scales showed acceptable internal consistency in the present sample, with reliability coefficients meeting or exceeding the commonly used α ≥ 0.70 criterion across the 10–16-year age range.

With regard to distributional characteristics, skewness and kurtosis values were generally within the recommended ±1 range. A slightly elevated kurtosis was observed for the General Self-Efficacy Scale; however, the deviation was small.

Group differences by sex and age were examined using two-way analyses of variance. Patterns were largely consistent with prior developmental research. Health-related quality of life (KIDSCREEN-10) was higher among boys than girls and also higher among younger adolescents compared with older ones. A significant interaction further indicated that the age-related decrease in HRQoL was more pronounced in girls than in boys. Older adolescents reported more problems in emotional and behavioral functioning (SDQ) consistent with the well-known increase in internalizing and externalising problems during mid-adolescence. Perceived stress (PSS) was also higher in older compared to younger adolescents. Both mindfulness (CAMM) and mentalizing ability (RFQY-5) increased with age, consistent with the progressive development of self-regulatory and socio-cognitive resources from early to mid-adolescence. Girls reported higher scores on both of these constructs, consistent with evidence that adolescent females report higher levels of emotional awareness and increased sensitivity to interpersonal cues. These convergent age and sex patterns support the interpretability of our measures and provide a coherent backdrop for the subsequent structural equation modelling analyses.

Consistent with theoretical expectations, health-related quality of life (KIDSCREEN-10) was strongly negatively associated with both the emotional and behavioral difficulties (SDQ) and the perceived stress (PSS), which implies that adolescents who reported more symptoms or higher subjective stress scores reported considerably lower overall well-being (Table 2). Furthermore, a small positive association emerged between HRQoL and the mentalizing ability (RFQY-5), indicating that adolescents who were more capable of interpreting mental states reported slightly higher well-being. The SDQ Total Difficulties score was strongly positively correlated with PSS and (in the opposite direction) strongly negatively correlated with mindfulness, which represents the well-known association between psychosocial difficulties, stronger negative stress experience and a reduced ability for non-judgmental awareness. Also, a parallel pattern was found for the PSS—mindfulness link, i.e., high negative stress experience was accompanied by reduced mindful awareness. In contrast to this, we found only small correlations between psychological resources—self-efficacy, mindfulness and mentalizing, which implies that although all these constructs reflect adaptive functioning, they represent different aspects of adolescents’ coping and regulatory capacities rather than a single psychological resource factor.

Overall, the correlational pattern indicates that stress and psychosocial difficulties form a tightly interconnected cluster, whereas self-efficacy, mindfulness, and reflective functioning contribute more independently to adolescents’ adjustment.

### 3.2. Structural Path Model of Stress, Psychological Resources, and Well-Being

**Figure 1:**
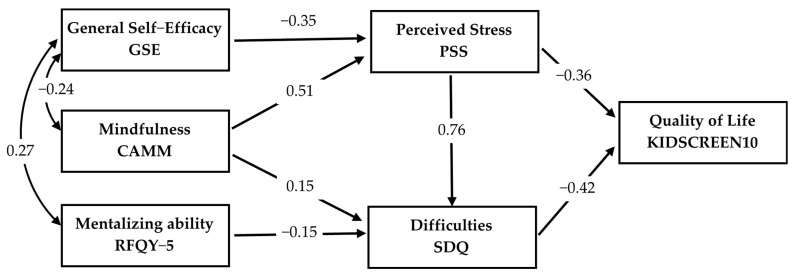
Final structural path model using observed variables linking psychological resources, perceived stress, emotional–behavioral difficulties, and health-related quality of life.

To examine how psychological resources, perceived stress, and emotional–behavioral difficulties jointly contribute to adolescents’ health-related quality of life, we estimated a structural path model including mindfulness (CAMM), general self-efficacy (GSE), and mentalizing ability (RFQY-5) as predictors of perceived stress (PSS) and emotional/behavioral problems (SDQ), which in turn predicted health-related quality of life (KIDSCREEN-10). We began with a theoretically informed full model and gradually removed all non-significant paths. This parsimony-oriented refinement was guided by modification indices, zero-order correlations, and theoretical considerations regarding the plausible direction of influence among constructs. The resulting final model therefore includes only statistically and conceptually meaningful pathways, offering a concise representation of the processes linking psychological resources to adolescents’ well-being (Figure 1; model fit: χ^2^(4) = 6.85, *p* = 0.144, CFI = 0.997, TLI = 0.990, RMSEA = 0.042, SRMR = 0.018).

A residual covariance between perceived stress and emotional/behavioral difficulties was retained (standardized value = 0.37), indicating that the two constructs shared substantial variance even after accounting for the three psychological resource variables. This residual association reflects the conceptual and empirical overlap between perceived stress and behavioral symptoms, consistent with their strong zero-order correlation (r = 0.66). Allowing their residuals to correlate avoids imposing an artificial causal direction in a cross-sectional design and yields a more realistic depiction of their empirically intertwined nature.

Within this final structure, both self-efficacy and mindfulness emerged as significant negative predictors of perceived stress, suggesting that these psychological resources operate mainly by reducing adolescents’ sense of unpredictability and overload rather than by directly shaping their emotional and behavioral adjustment. Mentalizing ability showed a small direct association with health-related quality of life but did not predict stress or emotional–behavioral problems, which is consistent with the minimal zero-order correlations observed between RFQY-5 and the other constructs. Reflective functioning showed a small direct link to adolescents’ health-related quality of life but was not significantly associated with perceived stress or emotional–behavioral problems in the final model. This suggests that, unlike self-efficacy and mindfulness, mentalizing ability may contribute to well-being through other pathways not captured by stress-related mechanisms.

Perceived stress in turn showed a strong positive association with emotional and behavioral difficulties, indicating that adolescents who experience their everyday lives as more overwhelming are also more likely to report internalizing and externalizing symptoms. Both perceived stress and emotional–behavioral difficulties independently predicted lower health-related quality of life, jointly accounting for a substantial proportion of variance in adolescents’ subjective well-being. Notably, none of the psychological resource variables had a direct effect on health-related quality of life once stress and emotional/behavioral problems were included in the model, suggesting that their influence is primarily indirect and mediated through adolescents’ stress appraisals and symptomatology.

To examine whether the structural model functioned similarly across key demographic groups, we conducted a series of multi-group invariance analyses for gender and age. Multi-group invariance analyses are summarized in Supplementary Table S1. For gender, model fit remained acceptable across all levels of constraint (ΔCFI = 0.011; ΔRMSEA = 0.007), but the slight decline in fit suggests that full structural invariance may not hold strictly. This indicates that some regression paths could differ modestly between boys and girls, although the overall pattern of associations was similar.

For age groups, all fit indices remained stable or slightly improved when equality constraints were imposed (ΔCFI = −0.009; ΔRMSEA = −0.008), supporting full structural invariance between younger (10–12 years) and older (13–16 years) adolescents.

Thus, while the model can be considered largely comparable across groups, age-related invariance was stronger than gender-related invariance.

## 4. Discussion

This research attempted to bring together three commonly studied psychological strengths (self-efficacy, mindfulness, and reflective functioning) into a single integrative model that links psychological resources to adolescents’ well-being through perceived stress and emotional–behavioral difficulties. Consistent with previous research, our findings indicate that psychological strengths such as self-efficacy and mindfulness are associated with better well-being primarily through lower perceived stress and fewer emotional–behavioral difficulties, rather than through direct pathways. Building on developmental and clinical perspectives, earlier research indicates that both psychological strengths and difficulties play an important role in adolescents’ emotional adjustment and well-being [15,53]. In line with this view, our model suggests that these strengths operate indirectly by shaping how adolescents experience, interpret, and cope with stress in their everyday lives.

Many studies have shown that self-efficacy is related to resilience and better coping abilities, less stress, and better emotional health [12,54]. Our findings match what has been found before: teenagers who see themselves as able to deal with difficulties have less stress, and this leads to less emotional and behavioral problems, and also better health and life quality. We also found that mindfulness works in the same way: teenagers who have better mindfulness skills have less stress. These results are similar to results from other studies [9,55,56] that indicate teenagers who are able to stay in the present moment tend to view everyday stressors as less overwhelming or uncontrollable. Reflective functioning in teenagers, measured by the youth RFQ, also leads to health and life quality in the same way, because it is related to low stress and low behavioral problems. These findings are like those from studies conducted in different populations that indicate adolescents who are better able to understand themselves and others show more effective emotion regulation [57,58].

In our final model, none of the examined psychological strengths (self-efficacy, mindfulness, or reflective functioning) were directly related to health and life quality. These findings are consistent with prior research. Well-being in teenagers is not just about their inner strengths, but also about how stress and trouble affect their daily functioning [59]. So, our results support ideas that look at strengths and weaknesses as steps in a process. The present model helps clarify how psychological resources support adolescents’ adjustment. These resources appear to function as indirect protective factors, mitigating the impact of stress and behavioral difficulties on well-being.

In the model, perceived stress is the center of the system, and directly predicts emotional and behavioral difficulties and health and life quality. This finding matches the literature about mental health in teenagers. Many studies state that perceived stress is one of the best signs of internal and external trouble in young people [14,26]. High stress has also been linked to intense negative emotional experiences, interpersonal difficulties, and poorer academic functioning [15].

The very robust path from perceived stress to emotional and behavioral difficulties (β = 0.76) emphasizes the central role of stress appraisal in adolescent adjustment. The present findings are in line with earlier studies identifying stress as a key correlate of both internalizing and externalizing symptoms in adolescence [15,60,61]. Perceiving life as stressful increases emotional and behavioral problems, which in turn undermine well-being. Consistent with this view, the residual covariance between stress and difficulties (−0.37) indicates that these processes are empirically inseparable and likely reciprocal: difficulties may also create stressful contexts, reinforcing vulnerability. This pattern highlights the limitations of cross-sectional designs and the need for approaches that capture dynamic interactions over time. In addition, the strength of the association raises the possibility of conceptual overlap between stress and symptom measures, which future research should address through careful operationalization and tests of measurement invariance. Taken together, our results highlight a process-oriented view: stress and problems do not serve as individual risk factors but as components of a system that influences health and quality of life. This view is also supported by longitudinal data indicating that emotional distress and associated processes usually represent reciprocal loops rather than a unidirectional pathway [62,63,64]. Such results emphasize the importance of multi-wave or cross-lagged designs to disentangle how these processes co-evolve and sustain each other in order to move beyond static snapshots toward a dynamic perspective on adolescent adjustment.

Emotional and behavioral troubles were a sign of low scores for health and life quality. This supports findings about how young people feel about well-being: it is related to what they want and feel, what they do, and how they get along with people. These findings have been used in different ways. A lot of studies state that how a teenager does on the SDQ is related to how sick and hurt they feel, how they are at school and with people, and how they feel about life [65,66,67]. Our findings show that health and life quality are closely related to emotional and behavioral strengths and weaknesses. This also places emphasis on the importance of identifying and addressing internalizing and externalizing problems early, before they escalate and begin to interfere more broadly with adolescents’ functioning.

Although mean-level differences emerged between boys and girls and between younger and older adolescents, the overall structural pattern of relationships was highly consistent across age groups and broadly comparable across gender. However, as indicated in Supplementary Table S1, the invariance tests showed a slight decline in fit indices for gender, suggesting that some structural paths may differ modestly between boys and girls. By contrast, full invariance was supported for age, indicating that the model structure functions equivalently from early to mid-adolescence. The similarities with the age groups, in particular, are important. The same score between old and young teenagers points out that the strengths measured in self-efficacy, mindfulness, and reflective functioning appear robust enough to be evidenced in younger as well as older adolescents. This is important because this study looks at early teenage years, when the abilities measured in our model are still new. Further, it should be remembered that the reliability and use of the different scales measuring strengths was mostly good from ages 10–16. This means that research with younger teenagers can use established measurement tools to understand how young people think about strengths in upper-primary school.

There are a few limitations in this study. Because the study used a cross-sectional design, causal relationships cannot be established. Although the proposed model derives from earlier conceptualizations and empirical data, alternative model specifications might emerge if different explanatory variables were considered. In the end, long or cross-lagged models are needed to see how stress, strengths, and emotional and behavioral problems grow and shrink over time, and how this predicts health and life quality. Second, all variables were assessed using self-reported data, which may introduce shared method variance across data points. Future research would benefit from incorporating multiple informants, such as parent or teacher reports, to complement adolescents’ self-reports. Third, even though it had good reliability, the study employed a brief measure of reflective functioning, which captures a general sense of mentalizing ability but may not reflect the full complexity of the construct. This may not show the full way of understanding oneself and others from the reflective functioning perspectives. Fourth, it should also be noted that the model focuses on health-related quality of life (HRQoL) rather than objective or clinical health indicators. Therefore, the findings should be interpreted as reflecting adolescents’ subjective well-being and perceived functioning. In this sense, health-related quality of life should be understood as a complementary outcome rather than a direct proxy for mental health. While HRQoL does not capture clinical symptomatology, it reflects adolescents’ subjective experience of functioning in everyday contexts, which is closely intertwined with, but conceptually distinct from, mental health. Lastly, the sample comes from one country, and therefore, similar research should be conducted using samples from other countries to replicate these findings.

Moving forward, research should examine how school, family, and peer-related stressors interact with psychological strengths to shape adolescent mental health, coping, and quality of life. Research could also look at how much teaching mindfulness, self-efficacy, or reflective functioning interacts with lowering stress and behavioral problems, and so increasing psychological health and quality of life. Such research could inform prevention programs aimed at promoting adolescents’ social and emotional well-being.

## 5. Conclusions

This research adds to a lot of research that tries to see how teenagers’ strengths work with stress to raise or lower emotions in different ways, and so health and life quality. The present research adds both conceptual clarity and empirical evidence to our understanding of how adolescents’ psychological strengths interact with perceived stress to shape emotional functioning and, ultimately, psychological health and quality of life. We have provided ideas about how five of these processes may work in one main model. The findings show that strengths found in self-efficacy, mindfulness, and reflective functioning are part of a working system. This system works on health and life quality not directly, but because of the relation these strengths have with how teenagers work with stress and behavioral problems. This empirically established path was largely similar for boys and girls and across age groups. In the end, all of the different scales provided relevant and reliable data, even for the youngest teenagers. The findings combine with past research on strengths and weaknesses and show that it is important for prevention and research to see how to work with both strengths and weaknesses in young people. Strengthening these social and emotional resources may help mitigate the development of psychological difficulties and support adolescents’ psychological health in the short term. Therefore, interventions grounded in the present conceptualization and empirical findings may prove beneficial for adolescents experiencing psychological difficulties, both in terms of their short- and long-term psychological development and their ability to cope with perceived stress.

## Figures and Tables

**Table 1:** Descriptive Statistics, Reliability Indices, and Group Differences for the Study Measures (*n* = 395).

Variables	Items	Cronbach’s α	Skewness	Kurtosis	M (SD)	Group Differences
KIDSCREEN-10	10	0.80	−0.54	0.32	37.17 (5.98)	G < B **; Y > O **; Int **
Strengths and Difficulties Questionnaire (SDQ)	20	0.75	0.56	0.12	12.11 (5.61)	Y < O ***; Int *
Perceived Stress Scale (PSS)	14	0.86	0.15	−0.20	25.57 (9.12)	Y < O **; Int *
General Self-Efficacy Scale (GSE)	10	0.89	−0.63	1.09	29.71 (5.56)	ns
Child and Adolescent Mindfulness Measure (CAMM)	10	0.83	0.23	−0.40	16.88 (7.36)	G > B **; Y < O **
Reflective Functioning Questionnaire for Youth (RFQY-5)	5	0.77	−0.53	0.06	22.86 (4.08)	G > B ***; Y < O ***

Notes. G = girls; B = boys; Y = younger adolescents (10–12 years); O = older adolescents (13–16 years). ns = non-significant. Significant group differences are marked with directional symbols indicating which group scored higher (e.g., G > B * means girls scored higher than boys). Interaction effects (Int) indicate significant sex × age interactions. * *p* < 0.05; ** *p* < 0.01; *** *p* < 0.001. SDQ = Strengths and Difficulties Questionnaire; PSS = Perceived Stress Scale; GSE = General Self-Efficacy Scale; CAMM = Child and Adolescent Mindfulness Measure; RFQY-5 = Reflective Functioning Questionnaire for Youth.

**Table 2:** Spearman’s correlations between predictor, moderator, outcome, and control variables in boys and girls.

	KIDSCREEN10	SDQ	PSS	GSE	CAMM	RFQY5
KIDSCREEN-10	—					
Strengths and Difficulties Questionnaire (SDQ)	−0.63 ***	—				
Perceived Stress Scale (PSS)	−0.61 ***	0.66 ***	—			
General Self-Efficacy Scale (GSE)	0.38 ***	−0.38 ***	−0.44 ***	—		
Child and Adolescent Mindfulness Measure (CAMM)	−0.45 ***	0.59 ***	0.59 ***	−0.23 ***	—	
Reflective Functioning Questionnaire for Youth (RFQY-5)	0.16 **	−0.21 ***	−0.10 *	0.23 ***	0.00	—

Notes. SDQ = Strengths and Difficulties Questionnaire; PSS = Perceived Stress Scale; GSE = General Self-Efficacy Scale; CAMM = Child and Adolescent Mindfulness Measure; RFQY-5 = Reflective Functioning Questionnaire for Youth. * *p* < 0.05; ** *p* < 0.01; *** *p* < 0.001.

## Data Availability

The data that support the findings of this study are available from the corresponding author upon request.

## Supplementary Materials

The following supporting information can be downloaded at: https://www.mdpi.com/article/10.3390/children13010038/s1, Table S1: Fit indices for multi-group structural invariance analyses across gender and age.

## Abbreviations

The following abbreviations are used in this manuscript:

HRQoL	Health-related quality of life
SDQ	Strengths and Difficulties Questionnaire
PSS	Perceived Stress Scale
GSE	General Self-Efficacy Scale
CAMM	Child and Adolescent Mindfulness Measure
RFQY-5	Reflective Functioning Questionnaire for Youth
