# Supplementary material for: Psychological Resources, Stress, and Well-Being in Adolescence: An Integrative Structural Model
Source: Children (Basel). 2025 Dec 26;13(1):38. doi: 10.3390/children13010038 (PMC12839675; doi:10.3390/children13010038)
Supplement: Supplementary file 1 [file children-13-00038-s001.zip › children-4027509-supplementary.pdf]

Supplementary Table S1. Fit indices for multi-group structural invariance analyses across gender and age.

Grouping variable	Model	Description	χ^2	df	CFI	TLI	RMSEA (90% CI)	SRMR	ΔCFI	ΔRMSEA
Gender	M1	Configural – same structure, parameters free	15,371	8	0.991	0.974	0.068 (0.000, 0.119)	0.024	–	–
	M2	Constrained paths – all regressions equal	31.686	15	0.980	0.969	0.075 (0.038, 0.112)	0.042	0.011	0.007
	M3	Residual-constrained – dependent variable residuals equal	43.495	18	0.970	0.960	0.085 (0.053, 0.117)	0.083	0.010	0.041
Age (10–12 vs. 13–16)	M1	Configural – same structure, parameters free	40,614	18	0.973	0.965	0.080 (0.047, 0.113)	0.045	–	–
	M2	Constrained paths – all regressions equal	30.304	15	0.982	0.971	0.072 (0.034, 0.109)	0.045	−0.009	−0.008
	M3	Residual-constrained – dependent variable residuals equal	40.614	18	0.973	0.965	0.080 (0.047, 0.113)	0.045	0.006	0.008

Notes. All models were estimated using maximum likelihood (ML) estimation in JASP (version 0.18). CFI = Comparative Fit Index; TLI = Tucker–Lewis Index; RMSEA = Root Mean Square Error of Approximation; SRMR = Standardized Root Mean Square Residual. ΔCFI and ΔRMSEA values were used to assess invariance following recommended criteria for changes in fit indices [49,50].
